# A Simplified Diagnostic Classification Scheme of Chemotherapy-Induced Peripheral Neuropathy

**DOI:** 10.1155/2020/3402108

**Published:** 2020-01-25

**Authors:** Han-Wei Huang, Pei-Ying Wu, Pei-Fang Su, Chung-I Li, Yu-Min Yeh, Peng-Chan Lin, Keng-Fu Hsu, Meng-Ru Shen, Jang-Yang Chang, Chou-Ching K. Lin

**Affiliations:** ^1^Department of Neurology, National Cheng Kung University Hospital, College of Medicine, National Cheng Kung University, Tainan, Taiwan; ^2^Department of Obstetrics and Gynecology, National Cheng Kung University Hospital, College of Medicine, National Cheng Kung University, Tainan, Taiwan; ^3^Department of Statistics, College of Management, National Cheng Kung University, Tainan, Taiwan; ^4^Department of Internal Medicine, National Cheng Kung University Hospital, College of Medicine, National Cheng Kung University, Tainan, Taiwan; ^5^Department of Pharmacology, National Cheng Kung University, Tainan, Taiwan; ^6^Innovation Center of Medical Devices and Technology, National Cheng Kung University Hospital, College of Medicine, National Cheng Kung University, Tainan, Taiwan; ^7^National Institute of Cancer Research, National Health Research Institutes, Tainan, Taiwan

## Abstract

*Background and Objective*. The main purpose of this study was to develop a simple automatic diagnostic classification scheme for chemotherapy-induced peripheral neuropathy.

**Methods:**

This was a prospective cohort study that enrolled patients with colorectal or gynecologic cancer post chemotherapy for more than 1 year. The patients underwent laboratory examinations (nerve conduction studies and quantitative sensory tests), and a questionnaire about the quality of life. An unsupervised classification algorithm was used to classify the patients into groups using a small number of variables derived from the laboratory tests. A panel of five neurologists also diagnosed the types of neuropathies according to the laboratory tests. The results by the unsupervised classification algorithm and the neurologists were compared.

**Results:**

The neurologists' diagnoses showed much higher rates of entrapment syndromes (66.1%) and radiculopathies (55.1%) than polyneuropathy (motor/sensory: 33.1%/29.7%). A multivariate analysis showed that the questionnaire was not significantly correlated with the results of quantitative sensory tests (*r* = 0.27) or the neurologists' diagnoses (*r* = 0.27) or the neurologists' diagnoses (

**Conclusion:**

The results of our unsupervised classification algorithm based on three variables of laboratory tests correlated well with the neurologists' diagnoses.

## 1. Introduction

Chemotherapy-induced peripheral neuropathy (CIPN) is a major dose-limiting side effect of antitumor treatment. Persistent CIPN causes significant mobility impairment, functional morbidity, and has a serious impact on the quality of life [1]. The incidence of CIPN varies from 30% to 40% of patients receiving chemotherapy. The drugs most commonly associated with CIPN are platinum analogs, antitubulins, proteasome inhibitors, and thalidomide [2]. Among these agents, oxaliplatin is widely used for the treatment of colorectal cancers, and paclitaxel and carboplatin/cisplatin are commonly included in the chemotherapeutic regimens for patients with ovarian and endometrial cancers. These are common types of cancer in both men and women, and therefore, many patients suffer from CIPN. The most commonly reported form of neuropathy in CIPN is sensory predominant polyneuropathy, which mainly affects terminals of long nerves symmetrically.

Peripheral neuropathy is conventionally diagnosed through a combination of a subjective description of symptoms, manual neurological examinations, and laboratory tests. Although this process is useful in clarifying the types and severity of neuropathies, the process is time consuming and can be unfamiliar to doctors specializing in treating patients with cancer. In addition, some studies have indicated that laboratory tests are less sensitive than physical examinations [3], and the correlation between quantitative laboratory tests and clinical symptoms has not been well established [4]. Therefore, this diagnostic paradigm has not been widely used or only partially adopted in research on CIPN. On the other hand, semiquantitative questionnaires such as the patient-reported CIPN-20 (European Organization for Research and Treatment of Cancer 20-item module for CIPN) [5] are more widely used and recommended to assess sensory neuropathy [6] and for monitoring the clinical course of CIPN. However, notable interobserver disagreement and underreporting of the severity of CIPN have been shown with the use of questionnaires [7], and their role in the diagnosis and classification of CIPN is unknown.

In an effort to establish an integrated and efficient diagnostic classification scheme for long-lasting neuropathies in CIPN, we performed a comprehensive battery of evaluations on patients with the later stages of CIPN. The main goals of this study were (1) to investigate the relationships among subjective complaints and the results of physical examinations and laboratory tests, (2) to test whether CIPN could be classified naturally into subgroups according to the results of laboratory tests, and (3) to identify a small set of variables produced by the laboratory tests to classify CIPN, so that the process of classifying CIPN can be simplified and accelerated.

## 2. Methods

### 2.1. Subjects

This was a prospective cohort study. Patients were recruited from the oncology and gynecology outpatient clinics of the National Cheng Kung University Hospital. According to the pathological staging and physicians' decisions, the eligible patients were those with colorectal cancers who received oxaliplatin, and those with gynecologic cancers who received paclitaxel/carboplatin as the adjuvant chemotherapy (Supplementary [Supplementary-material supplementary-material-1]). The inclusion criteria were age older than 20 years, colorectal cancers or gynecologic cancers confirmed by pathology, a diagnosis of cancer more than 1 year after the start of adjuvant chemotherapy, and good general condition, both physically and mentally. The exclusion criteria were prior chemotherapy within 1 year; previous chemotherapy with known toxicity to the nervous system; existing hereditary or acquired peripheral neuropathies; major medical diseases such as diabetes mellitus, renal failure, liver cirrhosis, and unstable angina; and poor cooperation.

This study complied with the Declaration of Helsinki, and the study protocol was approved by the Ethics Committee of the National Cheng Kung University Hospital. Before entering the study, the purpose, potential hazards, and experimental procedures were fully explained to the patients, all of whom signed written informed consent forms. This study was registered with ClinicalTrials.gov, number NCT02481336.

### 2.2. Evaluation Procedures

The patients received three types of evaluation, including a questionnaire about their quality of life, semiquantitative scoring of subjective complaints, results of neurological examinations focusing on motor and sensory systems, and laboratory tests consisting of nerve conduction studies (NCS) and quantitative sensory tests (QST) of thermal and vibratory sensation.

The CIPN-20, a 20-item quality of life questionnaire ([Supplementary-material supplementary-material-1] of Supplementary Material), was completed under the supervision of a proprietary study nurse. The data collected from the CIPN-20 questionnaire were summarized as a single score. Neurological examinations to evaluate motor and sensory systems were performed. Two compound scores, i.e., the clinical version of total neuropathy score [8] and the modified inflammatory neuropathy cause and treatment sensory sum-score [9], were calculated.

Subjective sensory and motor symptoms were graded, and neurological examinations to evaluate motor and sensory systems of 4 limbs were performed. We used items including muscle power, deep tendon reflex, pin prick for pain sensation, Rydel-Seiffer graduated tuning fork for vibratory sensation, and two-point discrimination. Two compound scores, i.e., the clinical version of total neuropathy score (TNSc) [8] and modified inflammatory neuropathy cause and treatment sensory sum-score (mISS) [9], were calculated.

A set of routine NCS of four limbs were performed. Motor NCS and F-wave latencies were assessed on median, ulnar, peroneal, and tibial nerves, and sensory NCS were performed on median, ulnar, and sural nerves. Variables derived from the motor NCS included distal latency, amplitude, conduction velocity, F-wave latency, and H-reflex latency. Distal latency, amplitude, and conduction velocity were obtained from the sensory NCS. The results from both sides were averaged. In total, 27 variables were recorded for analysis.

The routine QST of thermal threshold and thermal pain thresholds in four limbs were evaluated sequentially in one session by using a commercial sensory and pain threshold evaluation system (Pathway, Medoc Advanced Medical Systems, Israel). The QST of vibratory sensation in four limbs were performed using another apparatus designed specifically to estimate the threshold amplitude of vibratory sense (VSA-300, Medoc Advanced Medical Systems, Israel). The results from both sides were averaged. In total, eight variables were derived from the QST for later analysis.

### 2.3. Neurologists' Diagnoses

Five well-trained neurologists acting as raters independently interpreted the results of NCS and made the following nonexclusive diagnoses: sensory polyneuropathy, motor polyneuropathy, entrapment syndrome, and radiculopathy. Entrapment syndrome mostly involves carpal tunnel syndrome and cubital tunnel syndrome, and radiculopathy includes both cervical and lumbosacral radiculopathies. The group diagnosis of all five neurologists for a patient was defined as being positive (+1) when three or more neurologists made the diagnosis, and negative (0) otherwise.

In order to investigate correlations between the results obtained by unsupervised clustering and those by the neurologists' diagnoses, a corresponding classification scheme based on the neurologists' diagnosis was proposed. At the first level, the patients were classified into those with normal and those with abnormal vibratory thresholds of the lower limbs (VL). Patients with normal VL were further divided into group G1 that had less than two of the four defined diagnoses, and otherwise group G2. The patients with abnormal VL were divided into group G4 that had motosensory polyneuropathy, and otherwise group G3.

### 2.4. Statistical Analysis

The sample size estimation was computed using the simulation-based Spearman correlation method based on 5000 simulations. The proposed sample of 20 per group provided approximately 80% power to detect a correlation of 0.5 when the null hypothesis was no correlation, with a one-sided type I error rate equal to 5%.

In order to reduce the dimension, i.e., the number of test variables, and find the most relevant variables, neurological examinations and laboratory tests of interest were classified using the classification and regression tree (CART) analysis [10]. In order to reduce the dimension, i.e., the number of test variables, and identify the most relevant variables, neurological examinations and laboratory tests of interest were classified using the classification and regression tree (CART) analysis [10]. The nonparametric approach, CART, was used to develop decision rules without making any assumptions about the nature of the underlying statistical model and relate the predictor variables (Supplementary [Supplementary-material supplementary-material-1]) to the outcome variables. The method involved the segregation of different values of classification test variables through a decision tree composed of progressive binary splits, and the optimal split was selected based on impurity criterion. Each parent node in the decision tree produced two child nodes, which in turn became the parent nodes that, in turn, produced their child nodes. This process continued until statistical analysis indicated that the tree fit without overfitting. In order to determine the optimal tree, the complexity parameter value was chosen based on the 5-fold cross-validation technique. To evaluate the accuracy of a classification with 95% confidence intervals, an internal validation technique, the .632 bootstrap method, was also used [11]. The rpart function of the software package R (rpart, version 4.1.10; The R Project for Statistical Computing) was then constructed to identify the major risk factors through a recursive partitioning process that divided the patients into groups.

A multivariate technique, canonical correlation analysis, was then used to examine the associations between any two sets of variables, for example, the variables generated from laboratory measurements and the semiquantitative scales derived from physical examinations. Canonical is the statistical term for analyzing latent unobserved variables that represent multiple observed variables. So, a canonical variate is the weighted sum of the variables in a set, and the canonical correlation coefficient measures the strength of association between two canonical variates. Spearman rank correlations were calculated to quantify the relationship between two sets of variables. All statistical tests were two-sided, and a *P* value of less than 0.05 was considered to indicate statistical significance. All analyses were performed using statistical software R 4.1-10 for Windows (https://www.r-project.org).

## 3. Results

### 3.1. Results of Evaluations

**Figure 1:**
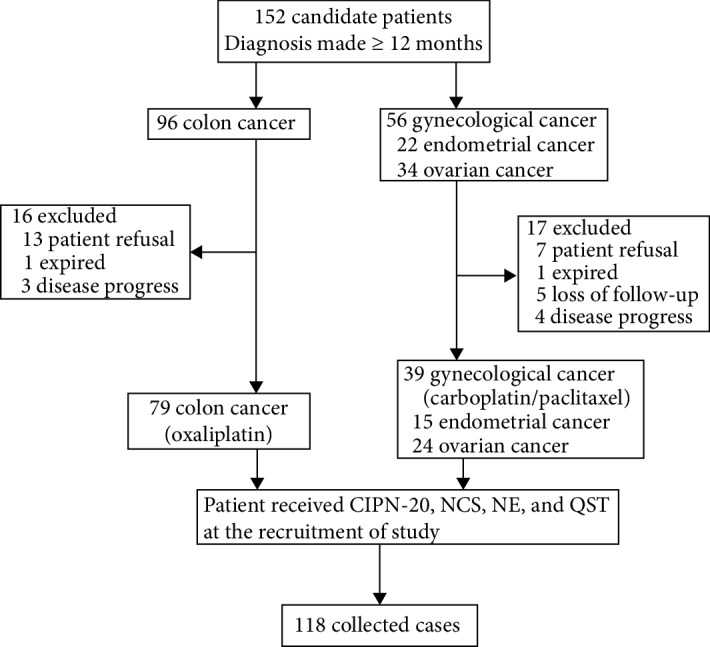
Flowchart of the study describing the recruitment of patients. CIPN-20: 20-item quality of life questionnaire specific for chemotherapy-induced polyneuropathy; NCS: nerve conduction study; NE: neurological examination; QST: quantitative sensory test.

From January 2016 to December 2017, 118 patients were recruited in this study (Figure 1). The clinical characteristics of these patients and the dosages of chemotherapeutic agents are summarized in Table 1.

The distribution of questionnaire scores is shown in [Supplementary-material supplementary-material-1] (Supplementary Material). Most of the patients reported having mild symptoms, including 67% with sensory symptoms and 68% with motor symptoms. Of note, 50% of the patients had moderate/severe autonomic symptoms. The results of neurological examinations and laboratory tests are summarized in Table 2. The mean duration from the start of chemotherapy to the evaluation date was 93 weeks (median: 52 weeks; standard deviation: 82 weeks). Overall, most of the mean values of NCS and QST data were within normal limits, except that vibratory, warm, and cold thresholds of the lower limbs were abnormal, and the sensory conduction velocity of both upper and lower limbs (threshold: 45 and 40 m/s, respectively) and the motor conduction velocity of the lower limbs were close to the threshold of abnormality (45 m/s). The latency of H-reflex was prolonged and abnormal. The results of QST in the lower limbs were more impaired than those in the upper limbs.

According to the neurologists' diagnoses based on the results of NCS and QST, 78 (66%), 65 (55%), 39 (33%), and 35 (30%) patients were diagnosed as having entrapment syndrome, radiculopathy, sensory polyneuropathy, and motor polyneuropathy, respectively (see [Supplementary-material supplementary-material-1] of Supplementary Material). In the canonical correlation analysis, the CIPN-20 was not significantly correlated with the neurologists' diagnosis (*r* = 0.20, *P* = 0.63) or QST (*r* = 0.27, *P* = 0.20). However, the QST showed a moderate correlation with the neurologists' diagnosis (*r* = 0.54, *P* < 0.001).

### 3.2. Classification of Patients and Reduction of Variables

**Figure 2:**
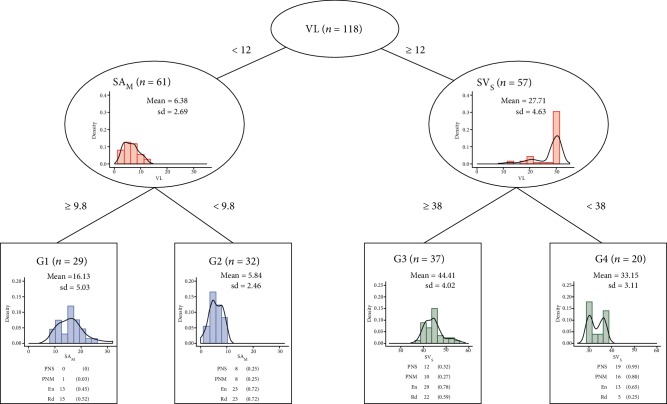
Clustering of patients into four groups using the CART algorithm with three variables derived from nerve conduction studies and quantitative sensory tests. SA_M_: the amplitude of compound action potential derived from the sensory NCS of the median nerve; SV_S_: the sensory conduction velocity of the sural nerve; VL: the vibratory threshold of the lower limbs; sd: standard deviation; PNS: sensory predominant polyneuropathy; PNM: motor predominant polyneuropathy; En: entrapment syndrome; Rd: radiculopathy.

Using hierarchical clustering, the patients were classified according to the distribution of 35 variables derived from the NCS and QST into four groups. Three variables, VL, the amplitude of compound action potential derived from the sensory NCS of the median nerve, and the velocity of the sensory NCS of the sural nerve (Figure 2), were chosen by the algorithm to make the classification. There were 14 misclassifications (11.9%) between the results of the unsupervised hierarchical clustering and those of CART with the three variables. Except for group G1 (colorectal/gynecologic cancers: 8/21), the other groups had more patients with colorectal cancers, i.e., group G2 (24/8), group G3 (27/10), and group G4 (20/0). The severity of neuropathy increased in the order G1, G2, G3, and G4.

Based on the classification results according to the neurologists' diagnosis, the overall correct rate of grouping by the CART algorithm was 78.8% (93/118, 95% confidence interval: 73.1%-88.3%). The classification accuracy was evaluated according to the averages of positive predictive value, negative predictive value, and the areas under the receiver operating characteristic curve, which were 0.89, 0.96, and 0.96, respectively. The results indicated that the model performed well.

**Figure 3:**
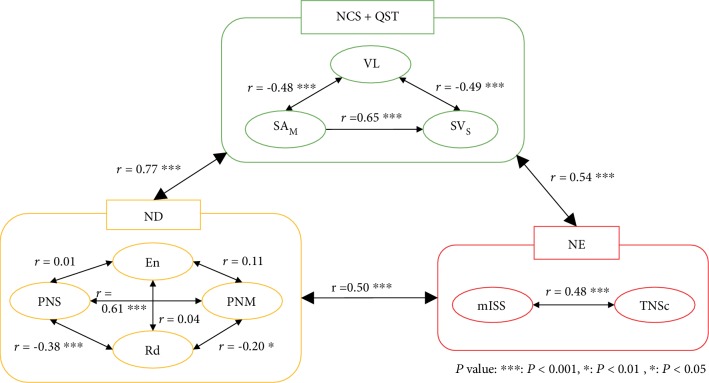
Canonical correlation analysis of relationships among the results of NCS and QST, neurologists' diagnoses, and scales of neurological examinations. Neurologists' diagnosis: neurologists' diagnosis based on nerve conduction studies; NE: neurological examination; VL: vibratory threshold of the lower limbs; PNS: sensory predominant polyneuropathy; PNM: motor predominant polyneuropathy; En: entrapment syndrome; Rd: radiculopathy; SA_U_: the amplitude of compound action potential derived from the sensory NCS of the ulnar nerve; F_P_: F-wave latency derived from the motor NCS of the peroneal nerve; TNSc: the clinical version of total neuropathy score; mISS: the modified inflammatory neuropathy cause and treatment sensory sum-score.

Figure 3 shows the relationships among the CART model, neurologists' diagnoses, and semiquantitative scales of neurological examinations according to the canonical correlation analysis. In intermodality analyses, all three modalities were well correlated, indicating that the CART model was a good representation of the neurologists' diagnoses. Of the neurologists' diagnoses, motor polyneuropathy correlated well with sensory polyneuropathy, implying that motor and sensory dysfunction usually coexisted even though the patients mostly only had sensory complaints.

## 4. Discussion

Scales derived from subjective complaints have been reported to be more sensitive than NCV in the sense that the positive rate was higher [12]. This conclusion may be based on the assumption that CIPN is equivalent to polyneuropathy. However, the specificity was unknown. Another evaluation method, QST, may be as sensitive as questionnaires of subjective complaints [13]. In addition, patients may adapt to chronic symptoms when neurological deficits persist. Thus, questionnaires of subjective complaints may not match the objective tests of NCS. Our results showed that the results of the CIPN-20 were not correlated with the results of QST or the neurologists' diagnoses.

The excellent match between the classification of hierarchical clustering and CART algorithm indicated that the distribution of the three variables chosen by the CART algorithm conformed to the global tendency of distribution of all variables. The good match between the classification from the CART algorithm and the neurologists' diagnoses implies that the classification by the CART algorithm may have important clinical implications. The classification rules according to the neurologists' diagnoses were not arbitrary, and VL was physiologically an indicator of large fiber neuropathy.

Most published studies of NCS in patients with CIPN have been incomplete, and only performed motor and sensory NCS in one nerve of a lower limb [12, 14]. Argyriou et al. reported that in patients treated with paclitaxel/cisplatin, the amplitude, but not the conduction velocity of sensory action potentials, reduced during the course of chemotherapy, and no significant changes were observed in the variables of motor NCS [14]. Our results (Table 2) also showed a similar tendency. Forsyth et al. [3] reported 37 females with breast cancer treated with paclitaxel and found that QST quantified the neuropathy but was less sensitive than physical examinations. In addition, they found that the most sensitive QST variable was VL, but that QST did not predict or identify subclinical polyneuropathy in any patient. Kroigard et al. [15] compared the diagnostic performance of skin biopsies to QST and NCS and concluded that the diagnostic sensitivity was the strongest with the skin biopsy followed by vibratory threshold in QST and then NCS. However, they stressed the importance of complete NCS in differentiating other etiologies. Taken together, these results are compatible with our findings, except that physical examinations and questionnaires had limited diagnostic sensitivity in our study.

To the best of our knowledge, no previous study has attempted to classify CIPN into subtypes. This is probably because CIPN was assumed to be equivalent to polyneuropathy in most studies. The major difference in the methodology of this study to previous studies is that we deliberately evaluated patients with a set of comprehensive tests. We found that the prevalence rates of radiculopathy (60.4%) and entrapment syndrome (70.8%) were much higher in the patients with CIPN, to the degree that we incorporated radiculopathy in the classification criteria of CIPN. Our results suggest that radiculopathy and entrapment syndrome may be the milder forms or residual abnormalities in NCS in the later stage of CIPN. Some more explanation.

Our classification model developed using the CART algorithm may have important implications for clinical practice. The objective classification of CIPN allows for the stratification of the prognosis and also tailors prevention strategies and treatment plans accordingly. The time and manpower required for laboratory tests can be greatly reduced when only three variables are needed.

## 5. Conclusions

A simple classification model of CIPN based on three laboratory test variables was constructed and was well correlated with the results based on neurologists' diagnoses. The proposed model may facilitate and accelerate the classification of CIPN.

## Figures and Tables

**Table 1:** Summary of clinical characteristics of the patients (*n* = 118).

Age (years), median (range)	56.8 (31~82)
Gender, number of cases (%)	
Female	76 (64)
Male	42 (36)
Body weight (kg), median (range)	58.4 (40.8~96.6)
Tumor type, number of cases (%)	
Colon	79 (67)
Ovary	24 (20)
Uterus	15 (13)
Mean dosage of chemotherapy agents, mg (sd^a^)	
Carboplatin (*n* = 32)	2507.98 (832.97)
Cisplatin (*n* = 7)	433.26 (47.84)
Oxaliplatin (*n* = 79)	1495.32 (307.46)
Paclitaxel (*n* = 39)	1724.28 (601.70)
Doxorubicin (*n* = 13)	376.20 (68.88)
Stage (I/II/III/IV)	
Colon	0/3/76/0
Ovary	6/5/10/3
Uterus	1/0/12/2

^a^sd: standard deviation.

**(a):** 

CIPN-20	27.30 ± 7.57
TNSc	3.59 ± 2.04
mISS	1.36 ± 1.42

**(b):** 

NCS		Distal latency (ms)	Amplitude (mV/*μ*V)	Velocity (m/s)	F latency (ms)
Median	MT	3.64 ± 0.86	7.70 ± 2.09	54.80 ± 3.87	25.77 ± 3.72
	SN	2.56 ± 0.45	8.09 ± 6.03	45.53 ± 7.57	
Ulnar	MT	2.48 ± 0.32	8.59 ± 1.77	58.07 ± 3.98	25.37 ± 3.73
				9.24 ± 3.52^a^	
	SN	2.03 ± 0.33	6.83 ± 4.15	47.02 ± 5.77	
Peroneal	MT	3.73 ± 0.65	4.51 ± 1.81	45.48 ± 3.46	46.65 ± 5.94
Tibial	MT	4.01 ± 1.20	12.60 ± 4.18	45.38 ± 3.73	46.08 ± 5.61
Sural	SN	3.31 ± 0.61	6.51 ± 5.01	43.17 ± 6.87	
H-reflex		33.55 ± 2.72			

**(c):** 

QST		CS	WS	HP
Thermal (°C)	UE	28.89 ± 1.82	36.93 ± 2.71	44.96 ± 2.38
	LE	26.40 ± 3.29	41.24 ± 2.45	46.35 ± 1.74
Vibration (*μ*m)	UE	4.10 ± 4.86		
	LE	16.69 ± 11.34		

^a^ The difference between nerve conduction velocities of the midforearm and cross-elbow segments. CIPN-20: 20-item quality of life questionnaire specific for chemotherapy-induced polyneuropathy; TNSc: the clinical version of total neuropathy score; mISS: the modified inflammatory neuropathy cause and treatment sensory sum-score; MT: motor; SN: sensory; UE: upper limb; LE: lower limb; QST: quantitative sensory test; CS: cold sensation threshold; WS: warm sensation threshold; HP: hot pain threshold.

## Data Availability

The datasets generated and analyzed during the current study are available in a public repository, https://data.mendeley.com/datasets/3kksgs4zc2/1.

## Supplementary Materials

Supplementary Materials Table S1 lists all the items in the CIPN-20 questionnaire, Table S2 shows the distribution of CIPN-20 scores of all patients, Table S3 shows the distribution of neurologists' diagnoses in groups classified by the CART algorithm, and Table S4 summarizes the NCS and QST results of normal and abnormal VL branches. Figure S1 shows the scheme of chemotherapy, and Figure S2 is a drawing for illustrating CART.
