# Supplementary material for: A Simplified Diagnostic Classification Scheme of Chemotherapy-Induced Peripheral Neuropathy
Source: Dis Markers. 2020 Jan 25;2020:3402108. doi: 10.1155/2020/3402108 (PMC7008270; doi:10.1155/2020/3402108)

Online-Only Supplementary Material

A simplified diagnostic classification scheme of chemotherapy-induced peripheral neuropathy

More description about the methodology and results of CIPN-20

Table S1 EORTC CIPN-20 items
Table S2 Distribution of CIPN-20 scores
Table S3 The distribution of neurologists' diagnoses in groups classified by the CART algorithm
Table S4 Summary of NCS and QST results of normal and abnormal VL branches

Figure S1 Scheme of chemotherapy
Figure S2 Illustration of classification and regression tree (CART)

More description about the methodology and results of CIPN-20

The questionnaire contains 20 items (Table S1) for assessing sensory (9 items), motor (8 items), and autonomic symptoms (3 items). Using a 4-point Likert scale (1: not at all, 2: a little, 3: quite a bit, and 4: very much), subjects indicate the degree to which they have experienced sensory, motor, and autonomic symptoms during the past week. The scores of the last two questions, asking about the sexual dysfunction of male patients and driving ability, were not counted for later analyses because some patients in Taiwan do not drive and the former was not applicable to the female patients. The data collected from the CIPN-20 questionnaire were summarized to give an overview of the patients' health-related quality of life and severity of chemotherapy-induced peripheral neurotoxicity. Based on the scores of symptoms, the severity of CIPN was classified into three categories, None: no discomfort (sensory score: 9, motor score: 7, autonomic score: 2); Mild: mild discomfort (sensory score: 10-17, motor score: 8-13, autonomic score: 3) and Moderate/severe: moderate or severe discomfort (sensory score $\geqslant$ 18, motor score $\geqslant$ 14, autonomic score $\geqslant$4).

The distribution of CIPN-20 scores is shown in Table S2. Ninety-five cases (80%) reported numbness, ranging from "a little" to "very much", in the lower limbs and 82 cases (69%) in the upper limbs. Only 16 cases (13.5%) reported thermal hyperalgesia, i.e. burning pain or shooting pain, ranging from "a little" to "very much", in the upper limbs and 15 cases (12.7%) in the lower limbs. A prickling or tingling sensation was reported in 46 cases (39%) in the upper limbs and 51 cases (43%) in the lower limbs. Hearing impairment and thermal insensitivity were not major complaints in this study. Ninety-one cases (77.1%) reported that hearing ability was similar before and after treatment, and 93 cases (78.8%) had no problem in distinguishing between hot and cold water. Fifty-five cases (47%) had difficulty in

climbing stairs or getting up from a chair due to weakness of their lower limbs, 48 cases (40.6%) had cramp in their lower limbs, and 48 cases (40.6%) had difficulty in opening a jar or bottle due to weakness in their hands, ranging from “a little” to “very much”. Most of the motor symptoms were described as “a little“, with 10% being “quite a bit” and 13.5% being “very much”.

Table S1 EORTC CIPN-20 items

Sensory scale (9 items)
1. Did you have tingling fingers or hands?
2. Did you have tingling toes or feet?
3. Did you have numbness in your fingers or hands?
4. Did you have numbness in your toes or feet?
5. Did you have shooting or burning pain in your fingers or hands?
6. Did you have shooting or burning pain in your toes or feet?
7. Did you have problems standing or walking because of difficulty feeling the ground under your feet?
8. Did you have difficulty distinguishing between hot and cold water?
9. Did you have difficulty hearing?

Motor scale (8 items)
10. Did you have cramps in your hands?
11. Did you have cramps in your feet?
12. Did you have a problem holding a pen, which made writing difficult?
13. Did you have difficulty manipulating small objects with your fingers (for example, fastening small buttons)?
14. Did you have difficulty opening a jar or bottle because of weakness in your hands?
15. Did you have difficulty walking because your feet dropped downwards?
16. Did you have difficulty climbing stairs or getting up out of a chair because of weakness in your legs?
17. Did you have difficulty using pedals?

Autonomic scale (3 items)
18. Did you have blurred vision?
    Please answer the following question only if you drive a car
19. Were you dizzy when standing up from a sitting or lying position?
    Please answer the following question only if you are a man
20. Did you have difficulty getting or maintaining an erection?

Items have been renumbered from the original EROTC CIPN-20

Table S2 Distribution of CIPN-20 (quality of life questionnaire) scores

	Sensory	Motor	Autonomic
**None**	10 (8%)	18 (15%)	25 (21%)
**Mild**	79 (67%)	80 (68%)	34 (29%)
**Moderate/severe**	29 (25%)	20 (17%)	59 (50%)

Table S3 Distribution of neurologists' diagnoses in groups classified by the CART algorithm

	Diagnosis			
**Group**	**PNS**	**PNM**	**En**	**Rd**
G1 (n=29)	0 (0)[#]	0.03 (1)	0.45 (13)	0.52 (15)
G2 (n=32)	0.25 (8)	0.25 (8)	0.72 (23)	0.72 (23)
G3 (n=37)	0.32 (12)	0.27 (10)	0.78 (29)	0.59 (22)
G4 (n=20)	0.95 (19)	0.80 (16)	0.65 (13)	0.25 (5)

[#] Percentage (number of patients)

PNS: sensory polyneuropathy, PNM: motor polyneuropathy, En: entrapment syndrome, Rd: radiculopathy.

Table S4. Summary of NCS and QST results of normal and abnormal VL branches

Branch		Normal VL		Abnormal VL	
		**Amplitude (mV/µV)**	**Velocity (m/s)**	**Amplitude (mV/µV)**	**Velocity (m/s)**
**Median**	**MT**	7.92 ± 2.13	56.04 ± 3.75	7.46 ± 2.03	53.48 ± 3.57
	**SN**	10.73 ± 6.46	48.14 ± 7.94	5.27 ± 3.95	42.74 ± 6.06
**Ulnar**	**MT**	8.71 ± 1.82	59.30 ± 3.64	8.45 ± 1.73	56.76 ± 3.93
	**SN**	8.45 ± 4.48	49.82 ± 5.38	5.09 ± 2.93	44.02 ± 4.55
**Peroneal**	**MT**	4.80 ± 1.89	46.60 ± 3.05	4.21 ± 1.70	44.28 ± 3.50
**Tibial**	**MT**	13.79 ± 4.11	46.68 ± 3.25	11.34 ± 3.9	43.99 ± 3.74
**Sural**	**SN**	8.39 ± 5.52	45.71 ± 6.18	4.50 ± 3.42	40.46 ± 6.56
**Vibration**	**UE**	2.36 ± 2.21		5.97 ± 6.10	
**(µm)**	**LE**	6.38 ± 2.69		27.71 ± 4.63	

MT: motor, SN: sensory, UE: upper limb, LE: lower limb.

VL: vibratory threshold of the lower limb.

Figure S1. Scheme of chemotherapy

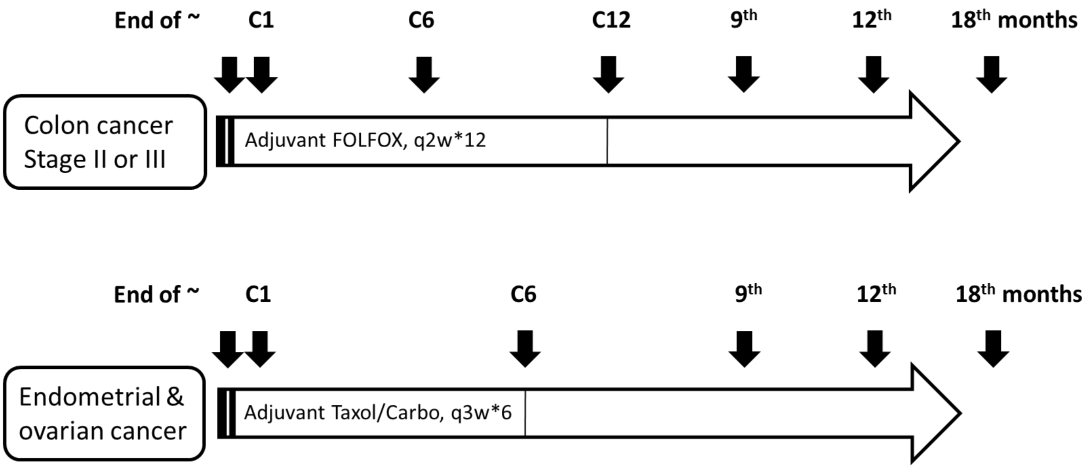

Figure S2. Illustration of classification and regression tree (CART). CART relates the predictor variables or parameters (X1, X2 and X3) to the outcome variables. Each (upper) parent node in the decision tree produces two (lower) child nodes according to the criteria value (a, b and c).

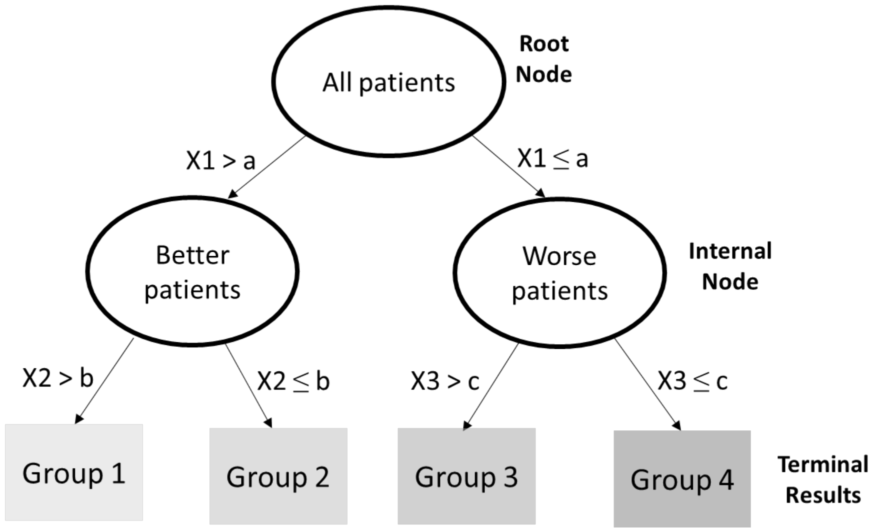

Supplement: Supplementary Materials — Table S1 lists all the items in the CIPN-20 questionnaire, Table S2 shows the distribution of CIPN-20 scores of all patients, Table S3 shows the distribution of neurologists' diagnoses in groups classified by the CART algorithm, and Table S4 summarizes the NCS and QST results of normal and abnormal VL branches. Figure S1 shows the scheme of chemotherapy, and Figure S2 is a drawing for illustrating CART. [file 3402108.f1.pdf]
